# Extended cubs’ care helping as a factor of reproductive success in a resident Golden jackal (*Canis aureus* Linnaeus, 1758) family group

**DOI:** 10.3897/BDJ.14.e191590

**Published:** 2026-10-27

**Authors:** Yulian Konstantinov, Boris Konstantinov, Stefaniya Kamenova, Ilya Acosta-Pankov, Nikolai Spassov

**Affiliations:** 1 Bulgarian Society for Regional Cultural Studies, Sofia, Bulgaria Bulgarian Society for Regional Cultural Studies Sofia Bulgaria; 2 Ultramarine Research Ltd, Aberdeen, Scotland, United Kingdom Ultramarine Research Ltd Aberdeen, Scotland United Kingdom; 3 National Museum of Natural History, Bulgarian Academy of Sciences, Sofia, Bulgaria National Museum of Natural History, Bulgarian Academy of Sciences Sofia Bulgaria

**Keywords:** Golden jackal, cubs’ care helpers, social organisation, bait-site monitoring, Danube bank (NW Bulgaria)

## Abstract

A resident Golden jackal family behaviour was studied over eight years (2017-2024) and especially over two successive post-birth seasons (May-September) in 2023 and 2024, utilising stationary bait-site camera-trap recordings on the Bulgarian bank of the Danube River. Earlier research on the cooperative breeding in golden jackals suggests that the cubs are typically cared for by the breeding pair and possibly by young helpers (1+/ 2+ years old). However, here we show that the presence of an adult, non-breeding helper (‘uncle’) can be systematic, helping the breeding female for several years, thus replacing the father, who, in our case, did not seem to contribute in the care for the young. Our observations also suggest repeated instances of food sharing by the non-breeding senior male adult, directed towards junior adults (last year’s jackals). Furthermore, food sharing behaviour amongst junior adults and towards the cubs was also recorded. Our data help revealing a more extended presence of ’helpers’ than is traditionally described in literature, with more than one or two junior adults remaining with the group to assist the breeding female during the denning period. This extended helping behaviour may contribute to the resilience of jackal family groups, particularly in contexts of pronounced hunting pressure, as is the case within our study area. We conclude that this specific behaviour, amongst other factors, may help to explain the demographic success in the European Golden jackal and with this, its significant recent expansion beyond its traditional habitats.

## Introduction

The current remarkable expansion of the Golden jackal (*Canis aureus* Linnaeus, 1758) (henceforth: jackal) from traditional areas of habitation has received substantial scientific attention (Tóth et al. 2009, Arnold et al. 2012, Rutkowski et al. 2015, Trouwborst et al. 2015, Spassov and Acosta-Pankov 2019). A predominant part of responsible drivers has been seen in various aspects of environmental change on the continent. For example, decreases in population densities across Europe of mega-carnivores, such as the wolf, has likely resulted in maeso-predator release (Levi and Wilmers 2012, Newsome and Ripple 2015, Trouwborst et al. 2015, Krofel et al. 2017). Land-use and climate changes have also been considered as important drivers, in particular deforestation, development of road networks and the increased availability of additional food sources due to the expansion of human activities (Spassov and Acosta-Pankov 2019 and references therein). Current ‘rewilding’ of abandoned agricultural land in Europe (Navarro and Pereira 2015, Perino et al. 2019, Tănăsescu and Constantinescu 2019), as well as the high dispersal potential of the species (Lanszki et al. 2018, Stronen et al. 2021, Kojola et al. 2024) can also contribute to the jackal populations’ ongoing success.

Against this background of remarkably successful expansion, a particular focus has been given to jackal social organisation. What has been increasingly demanding attention is the fact that, in the face of unrelenting extermination pressure in some countries, the animal not only resurges after culling, but continues to expand. This population resilience could be also explained by processes related to the in-group flexibility of animal social systems (Macdonald 1979). The case of the Golden jackal expansion in Bulgaria is particularly remarkable not only because of the very high intensity of lethal management (Stoyanov 2012, Acosta-Pankov et al. 2018), but also by the fact that it is currently the largest jackal population in Europe (Genov and Wassilev 1989, Kryštufek et al. 1997, Markov 2012, Stoyanov 2012, Markov et al. 2018).

This state-of-affairs has stimulated research directed at ‘secrets’ that jackal inner social life holds (see in particular: Custers et al. (2024)). In canids, cooperative breeding is commonly associated with socially monogamous mating systems, but where the non-breeding individuals could still provide alloparental care to the young under the form of food provisioning, for instance (Lukas and Clutton-Brock 2012). In Golden jackals specifically, the basis of the group is the breeding alpha pair, in which females and males share equal roles in pup-rearing and they are usually monogamous (Macdonald 1979, Moehlman 1987). In addition to the alpha pair, the family group also includes additional subordinate non-breeding adults sharing parenting (Macdonald et al. 2019, Pecorella et al. 2023).

Here, we present a documented case of alloparental care in the form of an extended helping by subordinate non-breeding adults (cf. Moehlman (1981), Moehlman (1987)), arguing that it is a critical mechanism for sustaining family groups in the context of pronounced lethal control. Our data from two consecutive field seasons (2023-2024) suggest that a strong non-breeding helper may stand as the critical factor for the resilience and resurgent capacity of the family group.

## Material and methods

We have deployed a comprehensive, long-term field-based approach consisting of observations of jackal social life in its day-to-day entirety, i.e. implying a longitudinal engagement with the same group by investing considerable time and observation efforts to gain an unprecedented qualitative depth into the social life of our focus family group. For this, we combined a manned, camera-trap directed towards monitoring a bait-site with extensive in-field visual/audial observations of jackal life at the core territory of a resident family group for a period of eight years (2017-2024). For the present study, we have used the data from the last two seasons only (2023-2024) as it is for these last two seasons that we have achieved sufficient certainty to guarantee the unambiguous individualisation of the family (breeding female, breeding male, non-breeding male).

**Study area**

Our study area is located on the Bulgarian bank of the Danube River (km 727, 43°50^'^54^''^ N, 23°24^'^48^''^ E). The area has seen a steady growth of its jackal population during the last 20-30 years, while previously no Golden jackals were recorded in this part of the country (Spassov 1989, Stoyanov 2012, Spassov and Acosta-Pankov 2019). The observation site was covered on three sides by dense growth of *Amorpha fruticosa* bushes, interspersed with deciduous trees (mainly poplar, beech and willow), the fourth side facing the river. Within the broader area, the *Amorpha* bushes frequently intertwined with bramble, wild vine and deciduous trees, forming a nearly impenetrable thicket for humans. The southern border of this strip of dense vegetation is a vertical face of a ten-to-fifteen metres high bluff. Beyond that and further away from the Danube River, the landscape extends into a mosaic of wild grass pastures, interspersed with belts of trees and thick undergrowth. The soil-composition of the bluff face and beyond it is of sedimental loess, allowing easy badger-sett digging, the latter amenable to secondary excavation by jackals. We attempted to replicate a waste-dump, typical around nearby villages and anglers’ tent-camps along the Danube bank, which jackals are actively exploiting.

**The bait-site**

The stationary bait-site was positioned close to the camp (~ 22 m), installed every year in April-May and removed by late August to mid-September, targeting specifically the denning and post-denning periods (May-August). For logistic reasons, observations in 2020 ceased in mid-July, while no fieldwork at all was carried out in 2022. Whenever possible, the pre-denning period (April-late May) has also been included. Details about in-field time and the jackal visiting-rate for the period 2017-2024 are provided in Table 1.

The bait-site itself represented a circular opening of approximately 6 m in diameter, located some 30 m from the birthing-den and at a distance to the river water’s edge from 1 to 50+ m relative to the widely fluctuating water-level.

**Camera-traps**

Since 2018, a camera trap has been strapped to a poplar tree overlooking the bait-site. The camera stood at a height of 1.5 m, 3 m away from the centre of the bait-site camera-covered space. Since 2021, the camera has been paired with a voice recorder. In 2023, a second camera was added, so that failures of one of the cameras would be compensated by the other, should such failures occur. The first camera used was a Victure Trail Game Camera 1080P, later replaced by two BlueGuard models (BG-310 and 662). Regardless of the model, all cameras were set to a combined picture and video mode. Video clips were initially 10 s in length, increasing to 30 s from 2021 onwards. The Passive Infrared (PIR) trigger interval was consistently maintained at 5 s.

**Bait**

The bait we used was composed of approximately 2 kg of rolled oats, soaked in water. To this basic ration, fish and fish innards were added, as well as a sliced half a loaf of bread, leftovers from the camp-table and all organic waste from cooking. The minimal ration was 2 kg of soaked oat-flakes mush. A maximum ‘mixed menu’ reached up to 6 kg (2/3 of an 8-litre pail).

The bait was deposited in the centre of the bait-site at around 18:00 h (DST-Sofia, UTC+3). The recording devices (cameras and voice recorder) were then strapped to an overlooking tree. An hour after sunrise, the devices were taken down for retrieval of overnight recordings. Processing and archiving of the material followed, together with the writing of diary notes about bait-content and quantity, weather conditions and all other contextually relevant information. All these data comprised a set of visual, audial and textual protocols, as parts of the data archive.

**Visitation**

The first stage of analysing the incoming visual data (stills and clips) has been to segment it into units, which we call visits. A visit begins when an animal enters the bait-site, stays there as a principal user and leaves it. The event can last from a minimum of a minute to about 20 minutes as a maximum. Overnight, the typical visiting rate oscillated between 10 and 12 visits of various family members, singly or in groups. At the same time, there were nights with zero visits, while the maximum number of recorded visits was 20. We have summed up bait-size visiting for the last two seasons in Table 2.

**Association**

The term ‘association’ here denotes the visits during which a senior adult (4+ yrs) would be present at the bait-site together with junior adults (1+/2+ yrs), cubs of the new litter (0+) or with both of these junior adult/juvenile cohorts.

**Care-giving**

We use the term in the sense of altruistic behaviour towards cubs and junior adults by individuals who are not the parents of such offspring of the same year or of the previous 1-2 years (Bergmüller et al. 2007).

The term ’care-giving’ here refers to two types of specific bait-site behavior:

- *Food-sharing*: when a family member of a hierarchically higher position shares food at the bait-site with one or more of the younger members;
- *Food-carrying*: when a family member carries food to another individual at the bait-site periphery (‘rim’) or carries food in the direction of the birthing den. The best example of this kind of behaviour, recorded by camera, is when a big morsel of food is being carried in the mouth (fish, part of a chicken carcass etc.) or most often - a slice of bread.

According to how food-sharing or food-carrying was done, we provided a scale for care-giving rating as follows:

- *nil* (grade 0): a hierarchical superior (’dominant’) chases away a junior adult or cub/s from access to food;
- *low* (grade 1): the dominant eats from the pile of food, but leaves enough for a sub-dominant who is/are waiting at the rim for the dominant to finish;
- *medium* (grade 2): the dominant allows a sub-dominant to eat together with them (‘nose-to-nose’ eating);
- *high* (grade 3): the dominant carries food and leaves it at the rim for the use of a waiting sub-dominant or carries food towards the den.

**Individualisation**

**Figure 1.**
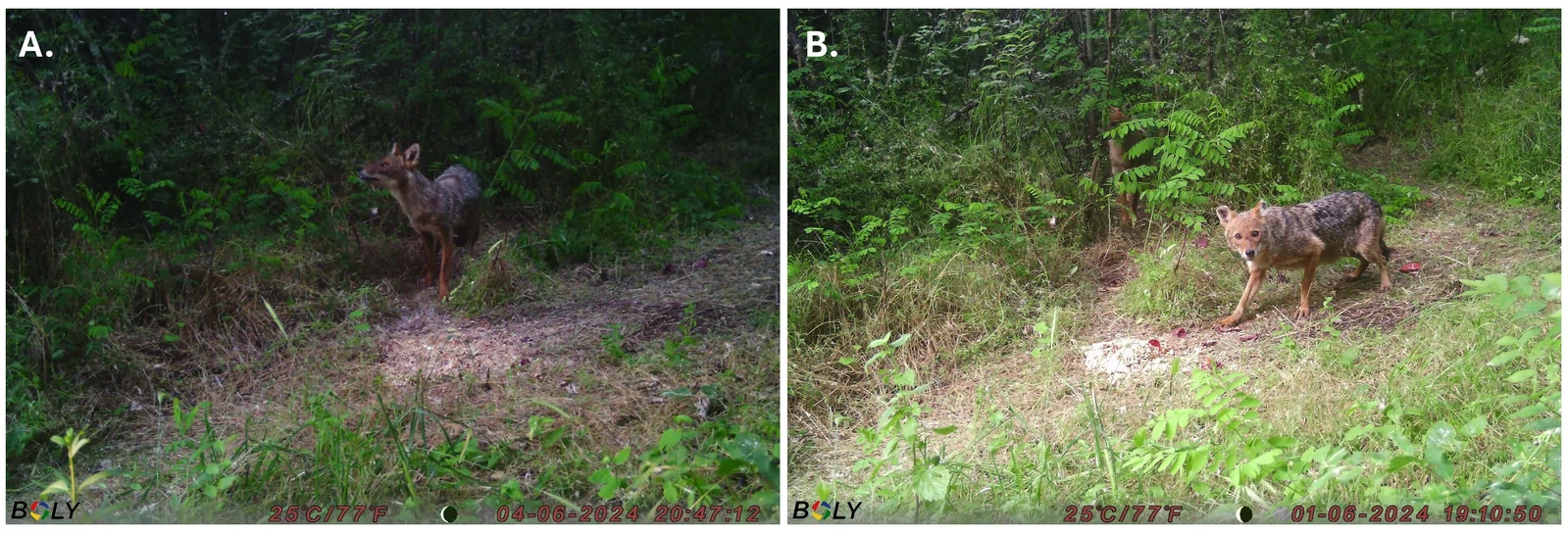
Ears as a distinctive feature for jackal individuation. **A** Unmarked (‘default’) pointed ears of a junior adult, 1+ year old; **B** Marked (‘clipped’) ears of a junior adult, 2+ years old.

Counting visits as well as associative/care-giving events involves the large issue of individualisation (’who’s who’). For dealing with this task, we have used a list of *distinctive features* (henceforth: DFs). The DFs recorded in reference to a particular individual, constitute their personal signature. We broadly divide DFs into marked and non-marked. Marked DFs denote exterior or behavioural features that stand out from a received (‘default’) reading of a jackal’s outer appearance or behaviour. As examples, concerning exterior features, illustrating a ‘default/marked’ binary, the form of the ears can be given (‘normal’ vs. ‘clipped’, ‘large’ etc.) as shown in Fig. 1 A and B.

**Figure 2.**
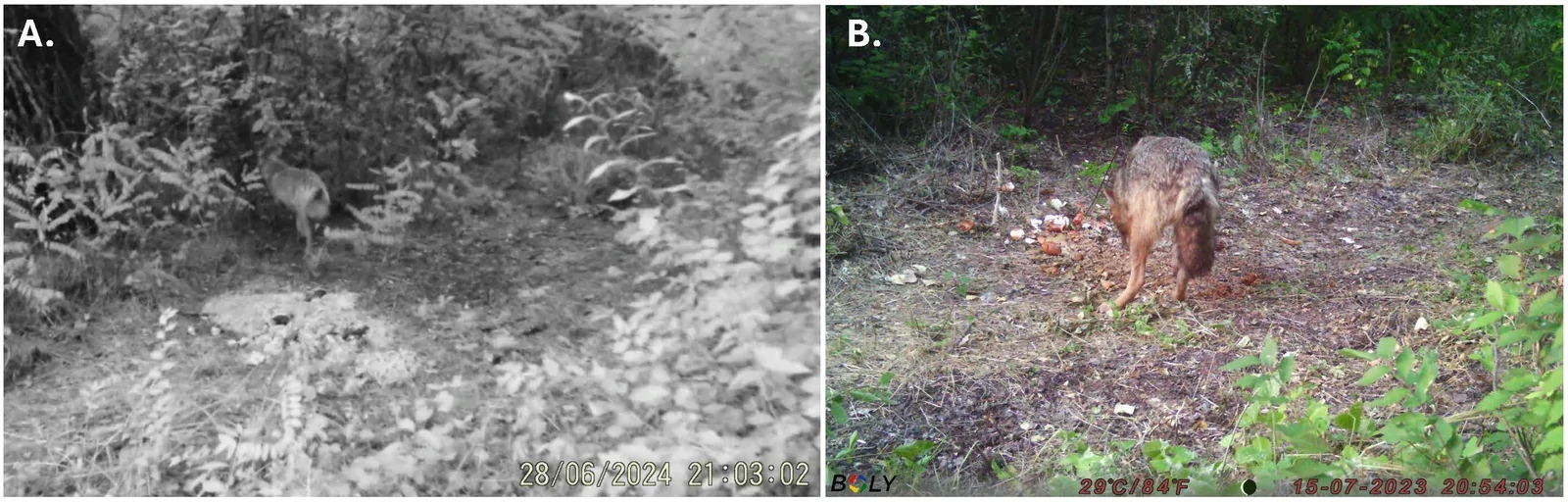
The tail as a distinctive feature for jackal individualisation. **A** The unmarked ’bushy’ tail with a swallow pattern of the breeding female; **B** The marked predominantly dark tail of the breeding male.

A helpful exterior DF (Fig. 2A and B), pointed out by Custers et al. (2024), is what can be called ‘tail-face patterns’. To their finding, we add two further features: (i) marked vs. unmarked tail faces; (ii) age-related features. Concerning (i), we treat ‘patterned’ tail-faces as unmarked (‘default’) tail features. Here, we follow the position that patterns are formed by dark hairs standing out against the lightening of the coat with age. A marked case is when such lightening does not take place in the tail of an adult animal and the latter remains dark.

**Figure 3.**
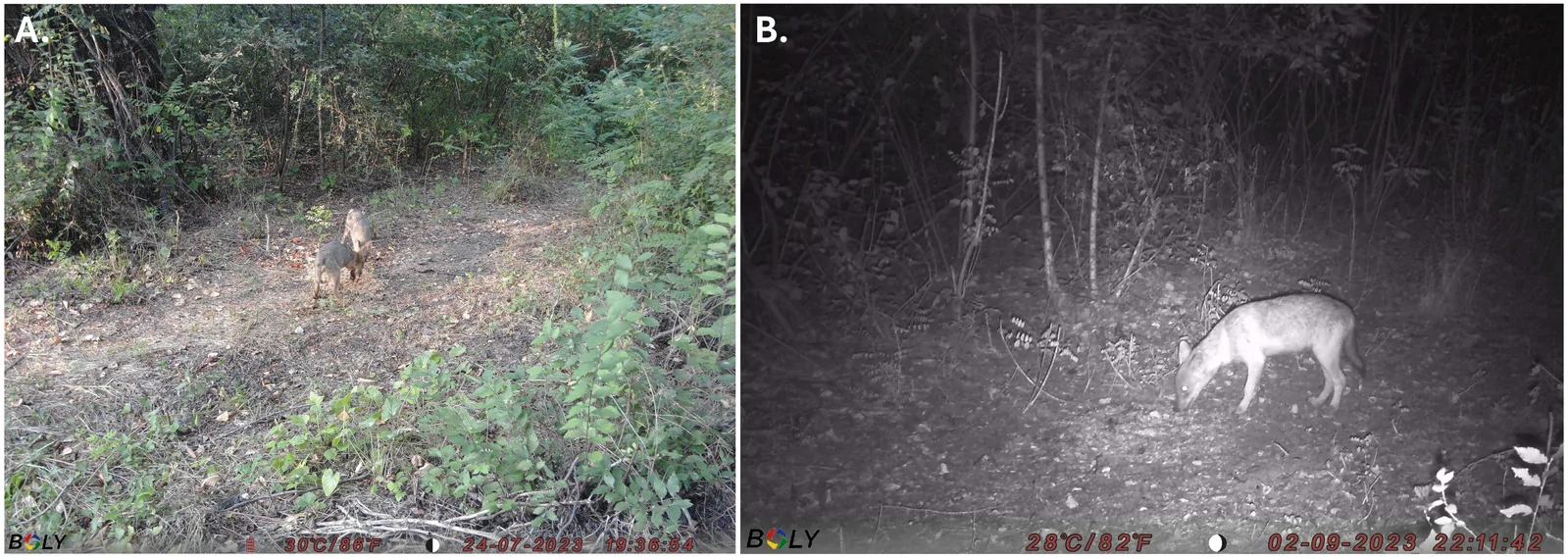
The tail as a distinctive feature for jackal cubs’ individualisation. **A** An unmarked thin tubular cub’s tail; **B** Side-view of unmarked dark tubular tail of a 3-month-old cub.

Regarding point (ii) made above, the ‘default’ juvenile tail is thin, tubular and hose-like in appearance (Fig. 3A). It looks longer in comparison with adult tails. No patterns are formed yet. In marked cases, a juvenile tail may look excessively long (Fig. 3B).

In summary, the following list of DFs has been used in this study:

I. Exterior

**Figure 4.**
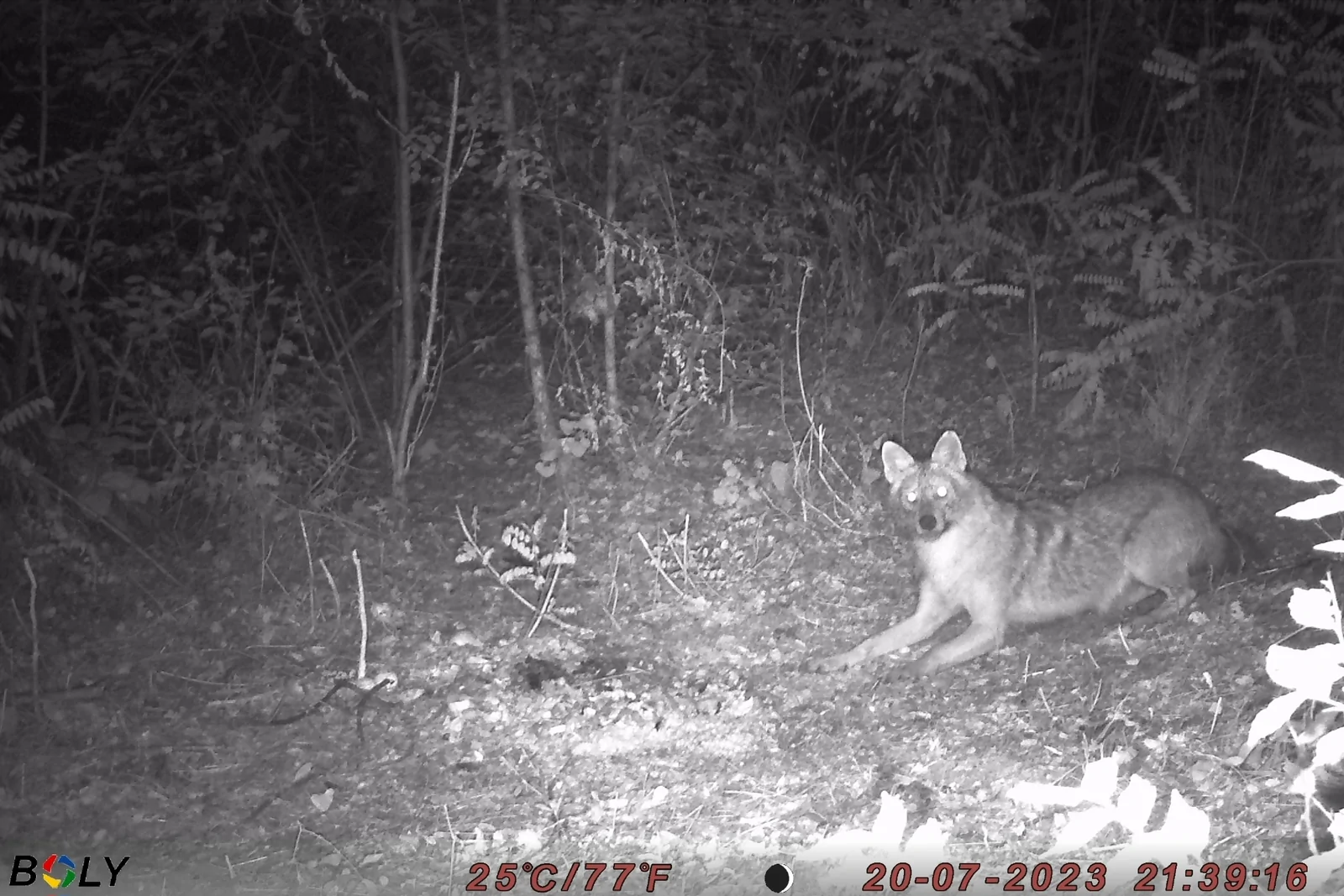
Marked ‘wolf-like’ muzzle features of the breeding male.

(a) Ears (pointed vs. rounded (’clipped’));

(b) Upward sloping hairy line in the genital area of an adult male (’brush’);

(c) ‘Calm’ vs. often bristling back ridge;

(d) Coat: dark (juvenile), paler (adult);

(e) Tail - diameter and length (e.g. small diameter and dark dorsal surface in juveniles vs. large, bushy, lighter hue tails in most adults; longish (reaching down below the hock in juveniles) vs. shorter (at hock level or higher in adults);

(f) Tail - individual markings on tail-face in adults (default); marked state: when a senior adult tail has retained – in a large part (2/3 from tip) - the dark hue characteristic of juvenile/junior tails;

(g) Muzzle - marked state: broad ‘wolf-like’ muzzle (Fig. 4).

II. Behavioural

(g) Time of bait-site visiting (marked: daylight evening, daylight morning; default: after sunset, before sunrise);

(h) Associative vs. non-associative visiting: an individual frequently/rarely/never appears in association with lower ranking family members; Associative visiting by members of the juvenile/junior adult cohorts (default);

(i) Care-giving vs. non-care-giving eating behaviour: during an associative event, the higher-ranking individual displays a high/middling/low/nil degree of food-sharing;

(j) Care-giving ‘helper’s behaviour’: an individual (except cubs) occasionally/often displays food-carrying towards the birthing den or to junior adults or cubs waiting at the bait-site’s periphery;

(k) Bait-site behaviour: entry/exit point, gait and posture (predominantly wary/fearful vs. predominantly confident);

(l) Camera-related behaviour: always facing camera (wary/fearful) vs. eating ‘back-to-camera’ (confident);

(m) Chemical marking: an individual frequently vs. rarely/never displays urine or faeces marking of the bait-site.

In Table 3 and Table 4, we summarise the exterior and behavioural characteristics of the family members, based on our observations since 2017 (for senior adults) and since 2021 (for junior adults).

**Computational Methods**

To visualise and compare the visitation patterns of subjects over the study duration, we employ a non-parametric Kernel Density Estimation (KDE). By utilising the existing data points, in our case - visitation records, we estimate the underlying probability distribution of events over time.

As the data are multimodal in nature, the band-width for each KDE was determined using the Sheather-Jones plug-in method (Sheather and Jones 1991) using the built-in stats package in R (v.4.x.x). It selects the optimal band-width by finding the value that minimises the Asymptotic Mean Integrated Squared Error (AMISE).

**Figure 5.**
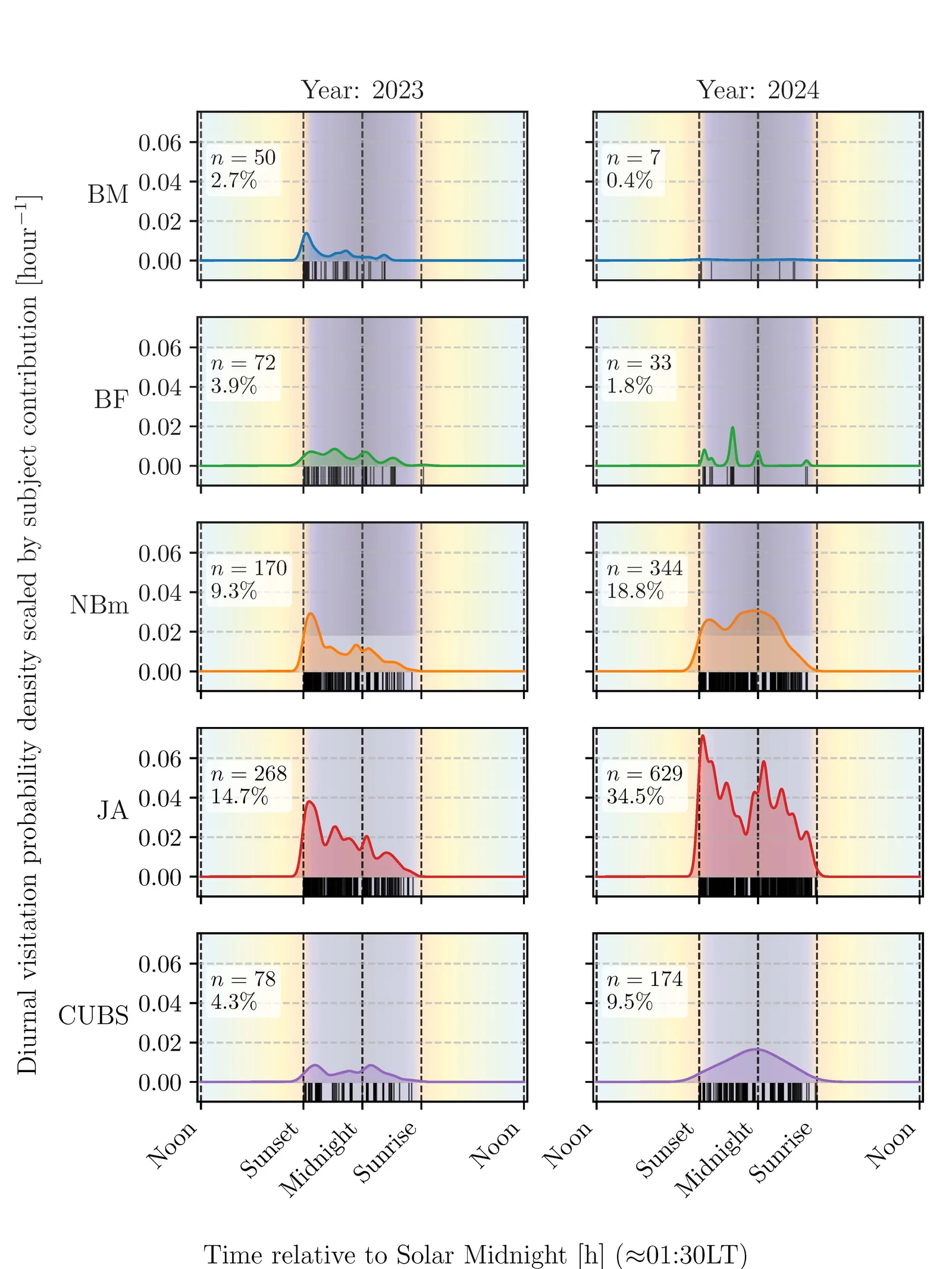
Diurnal timing of bait-site visits across subjects and years. Kernel density estimates (KDEs) show the distribution of visit times relative to the solar cycle, with midnight close to 01:30 h local time). The y-axis represents the share of total visits per hour, the area under each curve equalling that subject’s fraction of all recorded visits (2023–2024 combined, value provided in each subplot), enabling direct comparison of visitation intensity across panels. Peaks indicate increased activity during a specific timeframe with clear predominance of the ‘helpers’ (NBm and JAs). Rugs mark individual visits and n reports the number of visits contributing to each panel.

**Figure 6.**
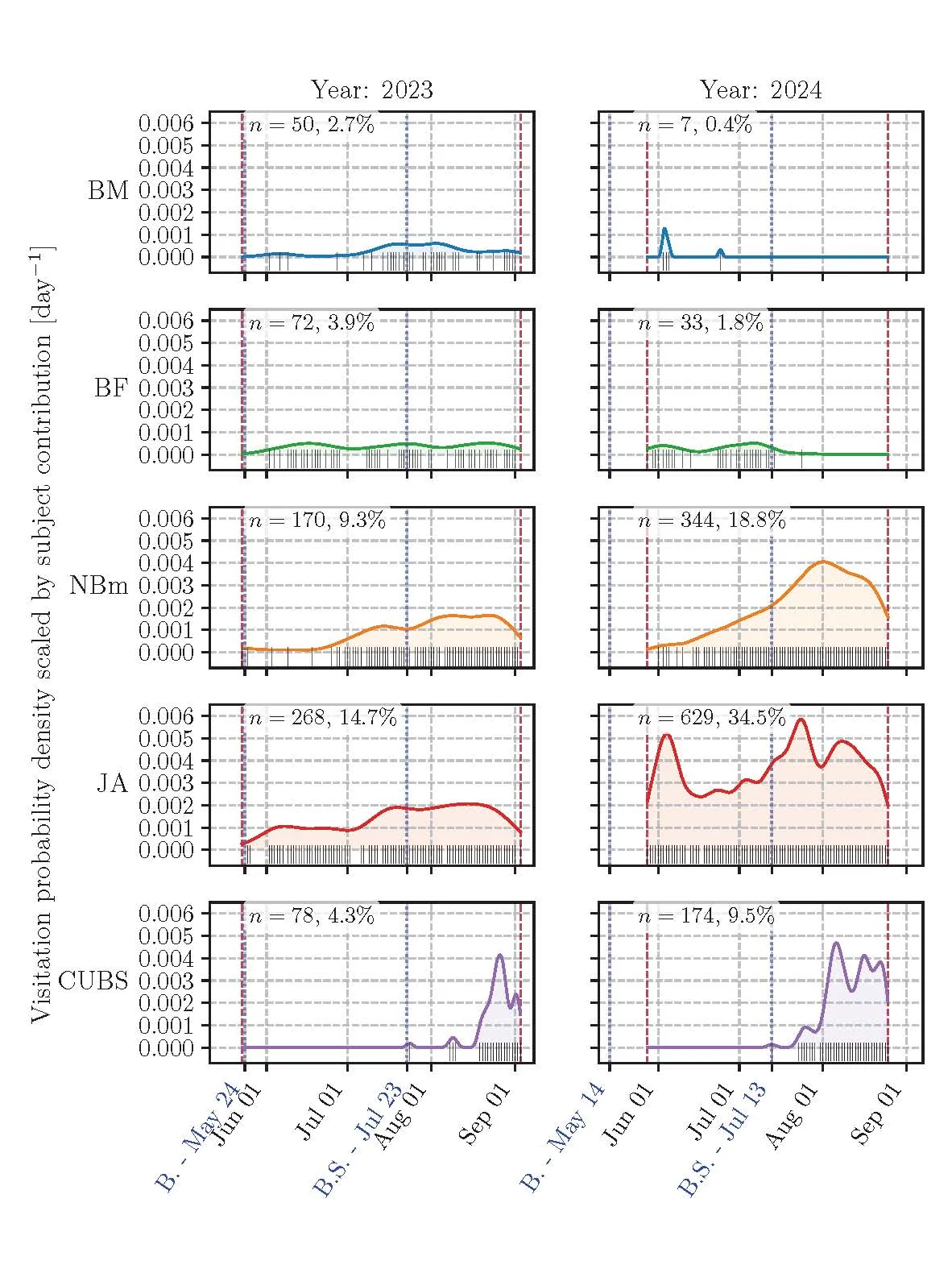
Seasonal timing of bait-site visitation across subjects and years. KDEs show visit dates over the observation window marked with vertical red dashed lines. The y-axis is the share of total visits per day, the area under each curve equalling that subject’s fraction of all recorded visits (2023–2024 combined, value provided in each subplot), so higher curves indicate more concentrated visitation and a larger overall contribution. We can clearly see the disappearance of the breeding male and breeding female around the middle of the 2024 observation period as well as the doubled presence of the non-breeding male and the significant increase in bait-site attendance from the non-breeding male succeeding those events. Rugs show individual visits and n gives the sample size per panel. Vertical blue dashed reference lines mark key seasonal dates shown on the x-axis.

To enable comparison between the relative activity of subjects at the bait-site in Fig. 5 and Fig. 6, each density curve was scaled by that subject's proportional contribution to the total number of events recorded for all years, obtained as, where it is the number of visits by a specific subject and is the total number of visits recorded for 2023 and 2024.

This scaling allows us to compare the KDE curves between different subjects and means that the y-axis now represents a scaled probability density function, where the area under all curves sums to 1 (equivalently 100%).

**Figure 7.**
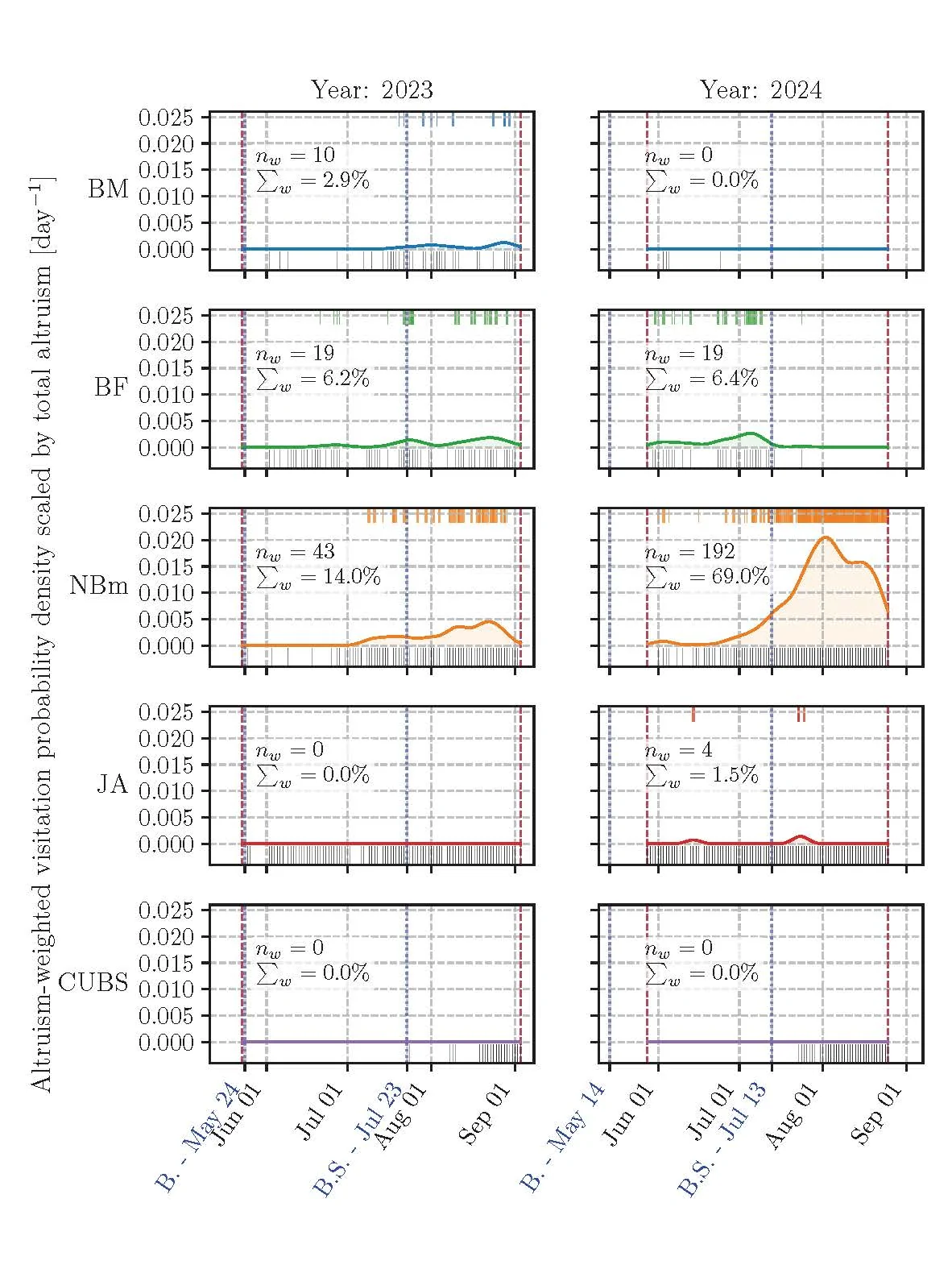
Seasonal timing of altruism-weighted bait-site activity across subjects and years. KDEs are computed as in Fig. 6, but each visit is weighted by altruism grade (0–3, grade 0 contributes zero weight) and then scaled so the y-axis represents share of total altruism per day. Consequently, the area under each curve equals the subject’s share of all recorded altruism (2023–2024 combined, sum of weights provided in each subplot), so this figure highlights when care-giving is expressed rather than simply if an actor is present. Panels separate high visitation with low observed care-giving (i.e. the juniors) from visitations with observed care-giving such as in the case of the non-breeding male. Bottom rugs show all visits, “curtains” at the top show only non-zero-weight visits with stroke width proportional to altruism grade. n reports non-zero-weight visit count.

In Fig. 7, where we wanted to incorporate the behavioural significance of each visitation, we applied the same scaling technique, but instead of scaling by proportional contribution, we scaled by the summation of altruism (care-giving) scored for each subject, compared to the total altruism scored for all subjects across both years.

Further in Fig. 6, we modified the standard KDE by introducing a weighting system, based on observed altruism. Events where an altruism score was not assigned or where the score was 0 were given a weight of 0, while events with low, medium and high altruism were assigned weights of 1, 2 and 3, respectively in a scale that increases linearly. The y-axis in Fig. 7, therefore, represents a scaled and weighted probability density function, where the area under all curves sums to 1 (equivalently 100%).

Assigning weights allows us to emphasise the quality of the altruism displayed by a subject. The question of how to assign weights is a subjective one and the choice of a linear scale is a compromise between simplicity and the need to capture the nuances of altruistic behaviour. Reproducing graphs with an exponential scale did not yield significant changes to the shape of the curve or the area underneath.

All KDEs are fitted with a rug plot at the bottom, showing individual events for each subject with the goal of giving the reader a sense of the quantity and density of the data that underpins the analysis. In KDE plots where we use weights, a rug plot at the top of the figure (henceforth 'curtains') is also present, where the width and height of each bar is scaled to the 'weight' of the datapoint.

To test the hypothesis that the non-breeding male's helping behaviour changed in response to the disappearance of the breeding pair, we constructed two generalised linear models in R (v.4.x.x) using the lme4 and ordinal packages.

First, to model the likelihood of any altruistic act of any grade occurring, we fit a generalised linear mixed model (GLMM) with a binomial error distribution. The binary response variable has values of 1 or 0 depending on whether altruism was observed. The primary fixed effect was a three-level factor for social phase. Phase 1 spans the duration where both parents (breeding male and breeding female) are present and defines as the period before 24 June 2024. Following that is Phase 2 when only the breeding female is observed as present, until 24 July 2024 when Phase 3 commences and both parents are presumed missing. Phase 1 is used as a reference level. To control for temporal changes in behaviour as the season progressed, we include the days since the observations have begun in a given year as a fixed effect. We also included the year of a study as a random intercept to account for any unmeasured baseline variation between the two study years.

Second, to model the change in the intensity of helping, we fit a cumulative link model (CLM) for ordinal data. The response variable was the four-level linear altruism grades (0, 1, 2, 3). The fixed effects (social phase and days since start of season) were the same as in the binomial GLMM. A random intercept was excluded from the ordinal model, as the two years of data did not meet the minimum level requirement for a grouping factor in the ordinal package.

## Results

**Individualisation**

According to the list of DFs in **Section Individualisation**, during the ‘23 and ‘24 seasons, the resident jackal family ‘hosting’ the bait-site at the Research Station has consistently maintained the following post-birth composition: three senior adults above 4 years old (one breeding male [Bm], one breeding female [Bf] and one non-breeding male [NBm]; four junior adults aged 1-2+ years old [JAs] and four cubs aged under 1 year old [Cbs]).

At the beginning of each of the two selected seasons (late May), the size of the group was of seven members - three senior adults plus four juniors. From late July on, the new litter increased this size by four cubs. At the beginning of next season, numbers were back to seven members. Dispersal or loss was attributed to the junior adults and cubs, as the three senior adults remained the same. Secure individuation could be claimed for the senior adult cohort.

**Overnight Visitation Rate**

We have estimated the visiting rate of the three senior adults (Bf, Bm and NBm), as well as of the four junior adults and the four cubs. Visiting of the senior actors has consistently been individual within their cohort. In other words, combined visits of these actors have not occurred in '23-'24. As different from them, members of the two other cohorts (junior adults, cubs), typically visit in pairs or groups of three or even four actors.

The results of the overnight visiting rate estimation are given below, in Fig. 6 as Kernel Density Estimates (KDE) subplots. On a shared x-axis, we plot time relative to the day/night cycle, centred on solar midnight. Each visitation is independently compared to the solar schedule of that day. An overlapping colour gradient indicates the time of day, with blue representing the night hours and yellow representing the day hours.

It is to be noted that the colour gradient itself is only representative of the day/night cycle for a single day. Solar event times may shift by as much as -7 to +3 minutes compared to the day/night cycle at the start and end of the study, respectively. On the scale of the portrayed data, this effect is trivial, but it should still be used only as a reference and not for absolute values. The final visualisation offers an intuitive depiction of each subject's temporal niche.

In 2023, we see Bm and NBm, favouring the time just after sunset. This is not contradictory with the statement made above and is simply indicative of their individual visitation patterns. More interestingly, NBm's visitation preference shifts from one aligning with the junior adults, to one aligning with the cubs in the second half of the study. This is indicative of a change in the NBm's role within the group, as he starts to spend more time with the cubs, possibly due to the absence of Bf.

**Season-long Visitation**

Fig. 5 illustrates the visitation density curves, each scaled by that subject's proportional contribution to the total visitation rate. Two red vertical lines mark the start and end of the study, while two blue vertical lines mark the start and end of the denning period.

In 2023, the visitation pattern shows prevalence of JAs visitation during the denning period (May-July). In August, this higher rate is preserved, while, at the same time, visiting by the NBm also rises, with the cubs making their first appearance during the second half of the month. The visitation rate for the breeding pair (Bm and Bf), is contrastively low, with a slight increase in the post-denning period, where they begin to display altruistic care-giving behaviour, as well, which is highlighted in Fig. 6.

Visitation patterns in the following season of '24 (Fig. 5) show marked differences. The most striking is the disappearance of Bm after the beginning of July and that of Bf later in that month. In contrast, visitation by JAs is very high all throughout the month and higher than that of the cubs. Likewise, NBm shows higher values in August than during the previous year. In summary, during the '24 season, the visitation rate of the non-breeding part of the group has roughly doubled compared to the previous year. The breeding pair shows diminished visitation in that season, both in terms of frequency and duration (duration is not a variable visible on the KDE plots, but a trend noted by the authors during their review of camera-trap footage).

**Season-long Care-giving**

In Fig. 7, we present the KDE of the same data as in Fig. 6, but scaled and weighted by the altruistic (care-giving) score of each subject. A curtain plot with markers proportional in size to the altruism score is also present.

Notably, when comparing the altruistic actors, in ‘23 vs. ‘24, we can see that the disappearance of Bm and Bf in '24 has led to a significant increase in the altruistic behaviour of the NBm, who appears to have taken over the role of the primary caregiver. This observation is examined further via models.

Results show that, following the disappearance of the Bm, the NBm was 3.1 times more likely (95% CL: 1.6 to 6.0, p < 0.001) to display altruistic behaviour of any grade in comparison to phase one and 6.8 times more likely (95% CL: 4.4 to 10.6, p < 0.001) with the disappearance of the Bf later that season. This demonstrates a clear compensatory response by the non-breeding male to the loss of the primary caregivers.

Beyond this response, our model identified that care-giving on the part of the NBm is not a static behavioural trait. Instead, the probability of altruistic behaviour is low at the start of the denning period (intercept = -2.5, p < 0.001), increasing steadily by approximately 2% each day as the season progresses (95% CI: 0.8% to 3.3%, p < 0.001). This is an indicator of caring behaviour adjusted to the development stage of the cubs, with a sudden increase at the end of the denning period as visible in Fig. 7.

Examining the grade of the care-giving displayed, we note a non-significant increase in the likelihood of displaying a higher grade of care-giving following the disappearance of the Bm (p > 0.05). With both primary caregivers gone, the quality of altruism (care-giving) changes significantly to 6.3 times greater than during the base period (95% CL: 4.1 to 9.8, p < 0.001).

In summary, we have measured a significant response by the NBm to the absence of the primary care-givers, both in terms of the frequency and the quality of the caring behaviour towards the cubs. In addition, our models and observations suggest that care-giving behaviour on the part of the NBm is not a consistent trait, but rather a developing response to the needs of the family.

In the context of the displayed altruism used as weighting for the visitation KDEs, it is worth noting that altruism (displayed as care-giving) in this study is only a measure of the tendency to share food at the bait-site and does not include behaviour at the den, such as nursing or regurgitating food for the cubs. This is a limitation of the study, as it has been focused on the bait-site and the path leading up to the den.

Another group that may suffer from this are the Junior Adults, who have been observed to carry food to the den during the denning period. Since no altruistic behaviour has been directly observed, this was scored at grade 0. Once the cubs emerge from the den, this activity ceases.

## Discussion

The previously unknown social behaviour in *Canis aureus* described by us can be attributed to the so-called cooperative breeding behaviour, in which some individuals help to raise offspring that are not their own (Bergmüller et al. 2007, Riehl 2013, Pecorella et al. 2023) and could probably be explained also by concepts that consider broader behavioural phenomena, such as theories of cooperation (Bergmüller et al. 2007).

The data presented above show that adult jackal bait-site behaviour is disproportional in terms of visiting and altruistic behaviour of family members, defined as care-giving. In the course of seasonal bait-site observations (May-August), coinciding with the periods of denning and post-denning, the breeding male (Bm) shows both minimal bait-site presence, as also one that displays low care-giving behaviour. As it can be seen in Fig. 6 and Fig. 7, the breeding female (Bf) performs as regards both visiting rate and care-giving in values roughly twice those of Bm.

The performance of the non-breeding male (NBm) is in pronounced contrast to these two types of behaviour. In both visiting and care-giving terms, NBm displays very high values, particularly during Season ’24. This markedly exceeds the performance of the mother (Bf), as well as that of Bm, on both counts. Moreover, NBm’s performance is not only very high in the first half of the night (sunset to night-start), but also after the solar midnight. This finding needs to be read against the process of exhausting the bait-site resource. Being served an hour before sunset, the resource gets exhausted in the space of the first hours of use, i.e. until night-start or, at best, until the solar midnight (depending on the quantity and quality of the bait). The presence of Bf and the lower presence of Bm, rapidly diminishes after that, but, by contrast, NBm’s presence stays in considerably high values until night-end. Importantly, NBm’s care-giving performance is emphatically high.

Regarding the junior/juvenile cohorts, visiting and care-giving patterns structurally differ. Junior adults (JAs) visiting performance is the highest amongst all actors, staying so until dawn. Their care-giving behaviour is, however, low and mostly confined to sharing food amongst themselves and with the cubs, as well as occasionally carrying food up to the den. Once out of the den, the juveniles (Cubs), perform actively in visiting all through the night and up until dawn. When their curve for this variable is compared to that of the JAs, it can be seen that it peaks only after midnight. This finding points to their being kept away from the resource in its prime state, benefitting from it only by virtue of care-giving behaviour, overwhelmingly by NBm. In visiting terms, the treatment of the Cubs, thus, sharply differs from the junior cohort’s, who dominate the scene from the moment of serving onwards.

## Conclusions

The primary finding of this longitudinal study is the dominant standing of a senior adult male as a care-giving performer. As explained earlier on, care-giving performance is displayed to a varying degree by allowing a hierarchically inferior family member access to food. In a higher form of display, care-giving performance is displayed by an act of carrying food to the breeding female (Bf) or to the hierarchically inferior members.

It has also been shown that, when the context of the bait-site is concerned, it is dominated by JAs and NBm, in the latter case, in altruistic rather than purely visiting terms. Insofar that the bait-site in the given case is closely associated with a human habitation, senior adult care for the younger cohorts in a human-proximate context can be interpreted as an in-group strategy for deflecting anthropogenic threat. In operational terms, the presence of a senior non-parental adult at the bait-site during the danger-prone first half of the night, improves the protective capacity of the inexperienced juvenile cohort.

The reported findings simultaneously reveal that, in the recorded situation, it is not the breeding pair who exercise the main protection of the junior/juvenile cohorts (cf. Custers et al. (2024); for cases of a missing parent: Csányi et al. (2023)). Instead, this study shows that the main role of upbringing and protection and, consequently, of building up the resilience of the group, falls on the helpers’ segment of the group. This social system provides a high success rate for the survival of the young and is likely to contribute critically for the demographic success of the population.

## Figures and Tables

**Table 1.** Summary of seasonal jackal monitoring from 2017 to 2024.

Year	Station setting - Evacuation	Duration (days)	Camera-recorded visits	Notes
2017	10 Jun – 20 Aug	71	-	Pilot run, no camera installed.
2018	27 May – 11 Sept	107	282	1 camera.
2019	24 May – 22 Sept	121	606	1 camera.
2020	13 Apr – 12 Jul	59	320	1 camera.
2021	22 Apr – 1 Sept	131	548	1 camera and 1 sound recorder.
2022	-	-	-	No study conducted.
2023	22 May – 3 Sept	104	637	2 cameras and 1 sound recorder.
2024	27 May – 25 Aug	91	1230	2 cameras and 1 sound recorder.
**Total**		**684**	**3623**	

**Table 2.** Annual counts of family members’ visits recorded by age and breeding status for 2023 and 2024, with total values per year and per actor. Counts are based on camera data collected over 104 days in 2023 and 91 days in 2024.

Year / actor	Breeding female (4+)	Breeding male (4+)	Non-breeding male (4+)	Junior adults (1+, 2+)	Cubs (0+)	Total per year
2023 (104 days)	72	50	170	268	78	638
2024 (91 days)	33	7	344	629	174	1187
Total per actor	105	57	512	895	298	**1825**

**Table 3.** Distinctive features of the senior adult cohort.

**Family member**	**Morphological DFs**	**Behavioural DFs**
Breeding female (Bf)	Light hue; visible teats and active nipples (May-June); signature tail-face. According to many years of observations, the individual is (in 2024) at least 4 years old.	Average association with juniors; minimal bait-site association with cubs; low to medium grade of altruism (1-2); marked daylight visiting (pre-sunset); wary/fearful gait and posture; face-to-camera eating; rare urine marker
Breeding male (Bm)	Upward sloping rear abdominal line (‘brush’); often bristling ridge; 2/3 from tip dark tail, athletic build, powerful neck, massive head, wolf-like round face. According to many years of observations, the individual is (in 2024) at least 6 years old.	Minimal associative and altruistic behaviour (1); night-time eater; confident gait and posture behaviour; confident back-to-camera eater; frequent urine marker
Non-breeding male (NBm)	Very light hue; signature tail-face; signature ‘bushy’ tip of tail; absence of sex marking. According to many years of observations, the individual is (in 2024) at least 6 years old.	Exclusively dark-time visitor; pronounced association with juniors and cubs; medium to high grade of altruism (3-4); frequent carrying food to rim and den (‘slicing’); sporadic vocal conflicts with Bm (‘shouting’); confident behaviour (all parameters); frequent urine and faeces marker

**Table 4.** Distinctive features of the junior adult cohort.

**Individual**	**Morphological DFs**	**Behavioral DFs**
Junior adult 1 (‘Big Ears’ 1)	Big pointed ears; dark hue; long and all-dark tail, no tail marking visible; male sex marking. By 2024, 1-2 yrs old	Pronounced associative behaviour; occasional altruistic behaviour (1-3); both daylight and dark-night-time eater; wary/fearful behaviour (gait and posture, face-to-camera eating); rare urine marker at bait-site rim; positioning at centre of bait-site; helper’s behaviour evidenced by carrying slices of bread to birthing den
Junior adult 2 (‘Big Ears’ 2)	Big pointed ears; dark hue; long and all-dark tail, no tail marking visible. By 2024, 1-2 yrs old	Pronounced associative behaviour; occasional altruistic behaviour (1-3); daylight and night-time eater; wary/fearful behaviour (gait and posture, face-to-camera eating); rare urine marker at bait-site rim; typical waiting at bait-site rim; helper’s behaviour evidenced by carrying slices of bread to birthing den; benefactor of occasional altruistic behaviour by male brother (‘Big Ears’ 1)
Junior adult 3 (‘Round Ears’ 1)	‘Clipped’ ears; dark hue; long and all-dark tail, no tail marking visible; male sex marking. By 2024, 2-3 yrs old	Pronounced associative behaviour; rare altruistic behavior (1-2) in respect of sibling; occasional helper’s behaviour (carrying to den); daylight and night-time eater; pronounced wary/fearful behaviour (dragging to food-pile, face-to-camera eating); rare urine marker (bait-site rim); entry/exit by use of den-path; positioning at bait-site centre
Junior adult 4 (‘Round Ears’ 2)	‘Clipped’ ears; dark hue; long and all-dark tail, no tail marking visible; female gender marking	Pronounced associative behaviour; rare altruistic behavior (1-2), occasional helper’s behaviour (carrying to den); daylight and night-time eater; pronounced wary/fearful behaviour (dragging to food-pile, face-to-camera eating); rare urine marker (bait-site rim); entry/exit by use of river path; positioning at bait-site centre
